# Supplementary material for: Acculturation and Culturally Adapted CBT for Refugee Youth and Families—A Systematic Review
Source: J Child Adolesc Trauma. 2026 Jul 28;19(4):2193–214. doi: 10.1007/s40653-026-00947-7 (PMC13737252; doi:10.1007/s40653-026-00947-7)
Supplement: Supplementary file 1 [file 40653_2026_947_MOESM1_ESM.docx]

**Title**

Acculturation and Culturally Adapted CBT for Refugee Youth and Families—A Systematic Review

**Abstract**

**Background:** Refugee children are exposed to stressors and events that can severely impact their psychological wellbeing. Additionally, the acculturation process during resettlement can greatly shape their mental health. Acculturation-informed culturally adapted Cognitive Behavioral Therapy (CA-CBT) could provide refugee youth and families with psychological support that meets their complex needs. This review explores the effectiveness of acculturation-informed CA-CBT interventions for refugee youth and families, specifically in reducing trauma-related symptoms such as PTSD, anxiety, or depression as well as improving day-to-day functioning, effective coping skills, and resilience.

**Methods:** 17 databases were systematically searched between April 2022 and June 2022. Search strategies such as keyword searching, phrase searching, Boolean logic, and truncation were all used during this stage to ensure relevant studies and literature were found and included. Moreover, due to this topic’s niche nature, broad eligibility criteria were applied to ensure all relevant studies were included.

**Results**: 5 studies with acculturation-informed CA-CBT interventions for refugee youth were included. The studies indicate the use of acculturation-informed CA-CBT reduces trauma-related symptoms such as PTSD, anxiety, depression, and resource hardships while improving functioning, effective coping skills, treatment engagement, and resilience associated with acculturation. Comparing acculturation-informed CA-CBT with CA-CBT and treatment uninformed by acculturation was not possible due to study limitations.

**Conclusions:** Although these are not well-established interventions, growing evidence supports their use. This review demonstrates how 5 acculturation-informed CA-CBT interventions have been designed and implemented to meet the unique needs of refugee youth. Studies on this topic are scarce and further research is needed before concrete conclusions can be drawn.

**Keywords**

acculturation; culturally adapted cognitive behavioral therapy; refugee youth

**Introduction**

According to the United Nations High Commissioner for Refugees, 2023 witnessed a record number of displaced people, and this number has certainly continued to grow in 2024 due to new and ongoing wars, conflicts, and humanitarian crises around the globe (UNHCR, 2023a; UNHCR, 2023b). Of the estimated 114 million people forcibly displaced worldwide, 36.4 million are refugees (UNHCR, 2023a; UNHCR, 2023b). The UN defines refugees in the 1951 Refugee Convention as individuals who have fled their country of origin due to fear of persecution for reasons related to ethnicity, race, religion, nationality, membership of a specific social group, or political opinion (UN General Assembly, 1951). It is important to note that even though only one third of our global population is made up of children, they account for almost 50% of refugees around the world (UNICEF, 2022). Given that half of the global refugee population is under 18 years old, it is essential to explore psychological interventions tailored to the needs of refugee youth and families.

Understandably, when refugees move to new countries, their various needs related to safety, housing, employment, schooling, legal aid, etc., take precedence over their mental health needs (Davidson et al., 2010). However, research on refugee mental health highlights how refugees can develop mental health issues and psychopathology due to being forcibly displaced (Davidson et al., 2010). In addition to no longer having a stable and familiar home environment, refugee children are vulnerable to being exposed to violence, abuse, malnutrition, infectious disease, and the atrocities of war (Pacione et al., 2013; Vossoughi et al., 2018). As a result, refugee youth are likely to exhibit symptoms related to post-traumatic stress disorder (PTSD), trauma, depression, anxiety, grief, and loss (Pacione et al., 2013; Nasıroğlu et al., 2018; Halasa et al., 2020; Yayan et al., 2020). Furthermore, researchers have found that children dealing with loss and complex trauma are more likely to develop attention, social, emotional, and self-regulation difficulties (Lawson and Quinn, 2013).

Providing refugee youth and their families with effective psychological support and mental health interventions early can help prevent long-term psychopathological difficulties for refugee youth and aid them in the resettlement process (Taylor et al., 2022). Researchers also argue that mental health professionals, school staff, and government officials should make preventing more damage to their cognitive and emotional development a priority (Bean et al., 2007). Given the high severity of psychological distress experienced by refugee youth, addressing refugee youth and families’ mental health needs is a crucial part of the resettlement process (Bean et al., 2007; Davidson et al., 2010).

**Culturally Adapted CBT (CA-CBT)**

Refugee youth’s unique needs—dealing with the resettlement process, separation from family, severity of psychological distress, pre-migration trauma, post-migration stressors, etc.—often make it challenging for them to engage in typical mental health services (Alto et al., 2021). Other barriers like language, stigma, mistrust of governments and mental health providers, as well as cultural norms around managing trauma and mental health, also make it challenging for refugee youth and their families to access mental health services (Alto et al., 2021). In hopes of making interventions more accessible and effective for people of diverse backgrounds, researchers have opted to culturally adapt them. Researchers view Cognitive Behavioral Therapy (CBT) (specifically trauma-focused CBT) as the most effective evidence-based intervention for treating refugee youth with PTSD, anxiety, depression, and trauma symptoms (Lawton and Spencer, 2021). CBT is an evidence-based psychological intervention that involves a clinician and patient working together to create a treatment strategy to develop coping skills and change the patient’s maladaptive thinking and behavioral patterns (American Psychological Association, 2017). Trauma-focused CBT (TF-CBT) is a type of CBT that is designed to help patients with PTSD and trauma symptoms (Lawton and Spencer, 2021). The effectiveness of CBT interventions for refugee youth has been analyzed in several studies and systematic reviews (Tyrer and Fazel, 2014; Nocon et al., 2017; Lawton and Spencer, 2021; Genç, 2022; Taylor et al., 2022). Lawton and Spencer focus on CBT interventions for refugee youth and highlight how CBT reduced symptoms of PTSD, depression, and anxiety (Lawton and Spencer, 2021).

Although the vast majority of CBT research has focused on European or North American, middle-class participants, research on culturally adapted CBT (CA-CBT) has become increasingly prevalent (Naeem, 2019). CA-CBT is a modified version of CBT that considers factors such as the client’s cultural background, values, and language (Bernal et al., 2009; Hinton et al., 2012; Taylor et al., 2022). A growing number of studies have explored the effectiveness of culturally adapted interventions. A meta-analysis of 76 studies evaluating culturally adapted interventions found that the weighted average effect size was d = 0.45, meaning that there was a moderately strong benefit to culturally adapting interventions (Griner and Smith, 2006). Moreover, interventions conducted in the patient’s first language were twice as effective as English interventions (Griner and Smith, 2006). Additionally, a study aimed at supporting Iraqi refugee youth who were the children of torture survivors found that most of the participants did not significantly benefit from treatment as usual (CBT, TF-CBT, and other interventions) (Kira et al., 2015). However, after receiving a CA-CBT intervention called the multi-systemic, multi-component and multi-modal intervention model (MMMM), the participants’ PTSD, complex PTSD, depression, and anxiety symptoms decreased significantly (Kira et al., 2015).

Some researchers have also found limited support for culturally adapting interventions. In one study, Huey and Polo did not find significant outcome differences for ethnic minority youth receiving culturally adapted interventions versus unadapted interventions (Huey and Polo, 2008). However, they recognize the possibility that clinicians have been altering their interventions and treatments to the cultural requirements of their patients without recording it, making the comparison of culturally adapted interventions and standard interventions more complicated (Huey and Polo, 2008). Researchers have also argued that the lack of consistency and unsystematic ways in which researchers culturally adapt interventions makes it challenging to compare results for the adapted interventions’ efficacy (Bernal et al., 2009). Researchers have also suggested that cultural adaptations to evidence-based interventions are not always warranted and more research is required to understand how cultural differences impact treatment (Unterhitzenberger and Rosner, 2016). Nevertheless, the research on the effectiveness of CA-CBT for refugee youth is encouraging (Naeem, 2019; Taylor et al., 2022). This is a growing field and future research must build evidence by conducting further tests and trials that explore how specific adaptations to CBT and other variables (gender, acculturation, PTSD severity, etc.) impact participants’ responses to culturally adapted interventions (Naeem, 2019; Hinton et al., 2012).

**Acculturation**

Acculturation is a process that occurs when people from different cultural backgrounds interact with one another (Tan et al., 2020). Though sometimes used interchangeably, acculturation and assimilation are separate terms. According to Berry, assimilation, integration, marginalization, and separation/segregation are the four main strategies people use during the acculturation process (Berry, 1997). The extent to which cultural minorities adapt and function in a society with dominant cultures is usually related to the acculturation strategies they use (Tan et al., 2020). Given that acculturation is influenced by several variables and continues to change over time, researchers have proposed additional frameworks that capture the process’s dynamic nature (Titzmann and Lee, 2022).

The pre-migration trauma and psychopathological symptoms related to PTSD, depression, anxiety, etc. experienced by refugees are typically the focus of refugee-related research (Keles et al., 2018). However, the acculturation process that occurs during resettlement can also greatly impact refugee youth’s mental health and wellbeing (Keles et al., 2018). Studies have found that the acculturation process might be more difficult for refugees due to their high possibility of experiencing trauma (Buchanan et al., 2016). Acculturation-related challenges and stressors have been found to be associated with refugee youth’s mental health trajectories (Keles et al., 2018). During the resettlement process, refugee youth need to learn new languages, navigate new cultures, and adjust to their new living situations (Keles et al., 2018). Moreover, acculturation-related stress can come from both out-group interactions with members of the new community’s dominant cultures (e.g. difficulty communicating in new language, discrimination, racism, bullying, etc.) as well as in-group interactions with people who share the refugees’ cultural and migratory backgrounds (e.g. harassment for not upholding values and traditions of their culture of origin) (Keles et al., 2018).

Researchers have found ways to take acculturation into account when designing their interventions for refugee youth. In one study, researchers found that increased social support positively influenced the acculturation process of migrant youth (Oppedal et al., 2015). Researchers have also highlighted how acculturative stress could be seen as mediated by language, educational challenges, peer acceptance, social support, family support and socialization practices that are culturally specific (Barrett et al., 2003). Several researchers have decided to incorporate the resilience factors associated with positive acculturation in their culturally adapted interventions to make them even more effective for refugee youth and families.

**Rationale & Aims**

The purpose of this systematic review is to explore the effectiveness of acculturation-informed CA-CBT in reducing trauma-related symptoms such as PTSD, anxiety, or depression as well as improving day-to-day functioning, effective coping skills, and resilience among refugee youth and family populations. To the extent of the researchers’ knowledge, this review will be the first to assess studies with acculturation-informed CA-CBT for refugee youth and families. Systematic reviews (including ongoing reviews registered on PROSPERO by Diehl et al., Kaya et al., Morroni et al., O'Donnell et al.) explore topics related to the effectiveness of CA-CBT, CA-CBT and refugees, as well as acculturation and refugees (Mengistu et al., 2019; Lawton and Spencer 2021). However, existing reviews have not explicitly focused on acculturation-informed CA-CBT interventions for refugee youth and families. Ultimately, this systematic review topic has important implications for the individual and community health of refugees, public health, policy related migration and displacement, and future research on acculturation and CA-CBT. In light of the important health implications of this topic and the lack of related systematic reviews, this review is warranted.

Given that many refugee families experience acculturation-related difficulties during the resettlement process and post-migration, this review aims to investigate the current literature on CA-CBT and acculturation and explore how CBT interventions that have been culturally adapted and informed by acculturation impact refugees’ mental health and wellbeing. In doing so, this review strives to explore the potential mental health and wellbeing outcomes of acculturation-informed CA-CBT as well as contribute to the wider discourse on culturally adapting interventions to improve mental health support for refugee and migrant populations.

**Methods**

This systematic review was carried out in adherence with the guidelines set by the Preferred Reporting Items for Systematic Reviews and Meta-Analyses (PRISMA). Moreover, the protocol for this review has been registered with PROSPERO (ID: CRD42022324105).

**Eligibility Criteria**

Studies were included or omitted based on this review's eligibility criteria. Given the niche nature of this topic, broad eligibility criteria were applied to make sure that all relevant studies related to acculturation, CA-CBT, and refugee youth and families were included. The eligibility criteria for this review were as follows.

- Location: there were no location-related requirements for studies.
- Participants: participants of studies needed to be young people (under 18 years old) or adults (over 18 years old) in refugee families.
- Intervention: the intervention used could be any kind of culturally adapted CBT intended to improve refugee mental health or wellbeing that has been informed by acculturation in some way.
- Outcomes: all study designs (qualitative information and quantitative data) were eligible.
- Language: all sources included were written in (or translated into) English and published online.

**Search Strategy**

The aim of the search for this systematic review was to find published and unpublished studies that were conducted between 1950 and April 1, 2022 (see Table 1 for search terms). The researchers’ chose to include published and unpublished articles from a broad time frame of 1950 to 2022 to ensure historical comprehensiveness and capture foundational research in the development of this topic and all relevant literature that might have otherwise been missed. Moreover, this systematic review was conducted as part of dissertation research for a one-year master’s programme that was completed in August, 2022. Therefore, the study selection process for this review was conducted between April 14, 2022, and June 14, 2022 and completed before studies from 2023, 2024, and 2025 became available. Although the timeline of this systematic review limited the inclusion of any literature post-2022, the literature existing at the time of this review still provides relevant insight into the effectiveness of acculturation-informed CA-CBT interventions for refugee youth and families.

**Table 1** Search terms

“acculturat*” AND “refugee*” AND “migra*” OR “unaccompanied minor*” OR “displac*” OR “asylum*” AND “cognitive behavio* therapy” OR “CBT” OR “trauma-focused cognitive behavio* therapy” OR “TF-CBT” AND “cross-cultural” OR “culturally adapt*” OR “culturally sensitive*” OR “culturally enhance*” OR “culturally appropr*” OR “culturally inform*” OR “culturally ground*” OR “culture specific*” OR “culturally focus*” OR “cultur*” OR “adapt*” OR “ethnic*” AND “child*” OR “adolescen*” OR “young pe*” OR “young*” OR “teenage*” OR “infant*” OR “famil*” OR “parent*” OR “carer*” OR “caregiver*” OR “mum*” OR “mom*” OR “mother*” OR “dad*” OR “father*” OR “youth*” OR “minor*”

After the date range and search terms for this review were identified, seventeen scientific databases were searched. Search strategies such as keyword searching, phrase searching, Boolean logic, and truncation were all used during this stage to ensure relevant studies and literature were found and included. The seventeen databases searched are ASSIA (Applied Social Science Index and Abstracts), CABI (Global Health), Cochrane Library, EMBASE, Google Scholar, The Knowledge Network, ERIC (via EBSCOhost), the International Bibliography of Social Science (IBSS) Online, MEDLINE, OpenGrey, PsycINFO (Ovid interface), PsycINFO (via APA PsycNET), PubMed, Scopus, Social Services Abstracts, Sociological Abstracts, and Web of Science (Core Collection). In addition, snowballing techniques, such as searching reference lists (i.e., citation chaining) and reaching out to authors for access to studies and data, were used to identify additional studies. Moreover, to address the risk of publication bias, grey literature (OpenGrey, etc.) was also searched.

In accordance with PRISMA guidelines, the studies identified through this search were independently screened by two reviewers (Page et al., 2021). After relevant studies were identified, the studies were initially screened based on their titles and abstracts. If a study’s participants were not refugee youth and families, it was excluded. The remaining studies were then screened using full-text analysis and the eligibility criteria previously mentioned. If the studies did not meet the eligibility criteria then they were omitted from the review. The studies found through searching scientific databases, and studies found through other methods (e.g., citation searching), were screened in the same way. At the end of the search and screening process, a second reviewer checked all the included studies and was unsure about the inclusion of two studies (Ellis et al., 2013; Kira et al., 2015). The reviewers discussed their thoughts and partially resolved their disagreement. The reviewers decided to include Kira et al.’s study, because the core part of the proposed intervention appears to be a type of CA-CBT (specifically, continuous traumatic stress intervention (CCC-TF-CBT) (Kira et al., 2015). However, because both reviewers still disagreed on whether they should include Ellis et al.’s study in the review, a third reviewer was consulted. The third reviewer stated Ellis et al.’s study should be included in the review because the main intervention is a type of CA-CBT. As a result, Ellis et al.’s study was included in this review. For a detailed overview of the search and screening process, please see Figure 1. As indicated in Figure 1, it is important to note that none of the five studies included in this review were identified through supplemental methods (e.g., citation chaining, snowball searches).

**Quality Assessment (Risk of Bias)**

The quality assessment for this systematic review was carried out using the most recent version of the Mixed Methods Appraisal Tool (MMAT) (Hong et al., 2018). Hong et al. designed the MMAT to help researchers critically assess qualitative, quantitative, and mixed-methods studies at the same time and conduct parallel evaluations (Hong et al., 2018). The MMAT has been effectively utilized in several similar reviews (Troup et al., 2021; Moyes et al., 2022; Wichmann et al., 2022). The initial part of the MMAT asks researchers to filter all studies included in their review using two screening questions (Hong et al., 2018). If only one or neither of the screening questions are met, then assessing the studies any further is unfeasible (Hong et al., 2018). Once the studies have met the initial screening questions, the MMAT uses five criteria questions (that are specific to each study’s design) to assess the methodological quality of each study (Hong et al., 2018). Reviewers can then answer the criteria questions by responding with “yes” (the study’s author has clearly described the point in question), “no” (the study’s author has not discussed the point in question), or “can’t tell” (it is unclear whether the study’s author has answered the point in question) (Hong et al., 2018). The results of the MMAT quality assessment for this review indicate that the five studies included in this review would be categorized as high quality. For a detailed overview of the MMAT assessment of the studies included in this review, please see Table 2.

**Data Extraction**

The following data from studies included in this review was extracted: author(s), location, setting, design, sample characteristics, sample country of origin and status, sample size, aim(s), the intervention, the acculturation element, and key points (including outcome measures and results). The data extracted for this review has been displayed in Table 3. Given that this review includes quantitative, qualitative, and mixed-methods studies, the data has primarily been combined and displayed narratively (combining the data statistically was not applicable for this review). It is important to acknowledge that this review is vulnerable to potential sources of effect heterogeneity; differences in the way CA-CBT is informed by acculturation across studies, differences in the ways practitioners deliver CA-CBT to refugees, differences in the types of CA-CBT, refugees’ geographical differences, refugees’ cultural differences, and refugees’ personal differences could all be potential sources of effect heterogeneity.

**Results**

**Study Selection**

The PRISMA flow diagram (Figure 1) illustrates the steps of the search, screening, and study selection process. The study selection process for this review was conducted between April 14, 2022, and June 14, 2022. The database search provided a total of 1,675 results. Results were then screened based on their titles and abstracts. After that, the remaining 66 results were screened according to full-text analyses, and 61 results were omitted. Additionally, 36 studies were identified through citation searching. Five studies met the eligibility criteria for this review and were subsequently quality assessed. 13 studies that were screened and excluded from this review because they focused on CA-CBT for refugee youth but were *not* informed by acculturation, were flagged during the search process and included in Table 4. Two additional papers that were informed by acculturation were found, a practice paper by Alto et al. and a study protocol by Rosner et al. on CA-CBT interventions for refugee youth (Rosner et al., 2020; Alto et al., 2021). The 13 studies, practice paper, and study protocol are relevant to the topic and will be explored in more detail in the discussion portion of this review.

**Participant Characteristics**

The studies eligible for this review have varying sample sizes and participants from a variety of backgrounds. The studies’ sample sizes ranged from 27 participants to 229 participants, with a total of 523 participants. All the participants in the included studies were young people (between 11 to 18 years old).

Additionally, 262 participants are female and 258 are male (the information for 3 participants is missing). The

**Fig. 1** PRISMA flow diagram of article selection based on PRISMA guidelines (Page et al., 2021)

participants’ origin regions include Africa, Asia (East Asia, South Asia, Southeast Asia), Central America, the Caribbean, Europe (Central/Eastern Europe), the Middle East, North America (including Mexico), Oceania, and South America. Only two studies (Ellis et al., 2013; Kira et al., 2015) specified the participants’ country of origin (Somalia and Iraq). The participants in three studies (Ellis et al., 2013; Kira et al., 2015; Elswick et al., 2022) were all refugees. However, participants in two studies were a mixed migrant population (Beehler et al., 2012; Khawaja and Ramirez, 2019).

All the studies’ participants received the interventions through school/after-school programs. Four of the interventions were designed as school-based programs (Beehler et al., 2012; Ellis et al., 2013; Kira et al., 2015; Elswick et al., 2022). However, even though Khawaja and Ramirez’s intervention is not specifically a school-based program, it was administered in school and community settings due to emerging evidence on the benefits of offering interventions for culturally and linguistically diverse (CALD) adolescents in these settings (Khawaja and Ramirez, 2019).

Participants experienced traumatic grief and loss, community violence, abuse, assault, domestic violence, maltreatment, acculturative stress, behavioral issues, emotion regulation difficulties, discrimination, and bullying (Beehler et al., 2012; Ellis et al., 2013; Kira et al., 2015; Elswick et al., 2022; Khawaja and Ramirez, 2019). Four studies focused their interventions on improving participants’ symptoms related to PTSD, depression, anxiety, behavioral problems, and traumatic/complicated grief (Beehler et al., 2012; Ellis et al., 2013; Kira et al., 2015; Elswick et al., 2022). These studies also explored their interventions’ impact on participants’ functioning, effective coping skills, resource hardships (personal, material, social, etc.), and treatment engagement (Beehler et al., 2012; Ellis et al., 2013; Kira et al., 2015; Elswick et al., 2022). One study focused its intervention on the acculturation processes experienced by participants and fostering their resilience (Khawaja and Ramirez, 2019). All studies aimed to evaluate their interventions’ effectiveness in terms of improving participants’ mental health and wellbeing (see Table 3 for details).

**Study Methodologies**

The included studies used quantitative or mixed methods study designs. Each study involved a within- subjects comparison pre- and post-intervention. None of the participants in the studies were randomly selected. Furthermore, given that the studies only used one group of participants and all participants received the same intervention, no randomizing or blinding methods were used. All studies investigated the effectiveness of

| **Category of study designs**  **Table 2** Mixed Methods Appraisal Tool (MMAT) with results for this review (Hong et al., 2018) | **Methodological quality criteria** | **Responses** | | | |
| --- | --- | --- | --- | --- | --- |
|  |  | Yes | No | Can’t tell | Comments |
| Screening questions  (for all studies) | S1. Are there clear research questions? | ✔ |  |  |  |
|  | S2. Do the collected data allow to address the research questions? | ✔ |  |  |  |
|  | *Further appraisal may not be feasible or appropriate when the answer is ‘No’ or ‘Can’t tell’ to one or both screening questions.* | | | | |
| Quantitative non-randomized  Beehler et al. (2012) | Are the participants representative of the target population? |  | ✔ |  | Participants are a mixed migrant population. Even though refugees are included in this group, some of the participants might not be refugees and, therefore, not representative of the target population. |
|  | Are measurements appropriate regarding both the outcome and intervention (or exposure)? | ✔ |  |  |  |
|  | Are there complete outcome data? | ✔ |  |  |  |
|  | Are the confounders accounted for in the design and analysis? | ✔ |  |  |  |
|  | During the study period, is the intervention administered (or exposure occurred) as intended? | ✔ |  |  |  |
| Quantitative non-randomized  Ellis et al. (2013) | Are the participants representative of the target population? |  | ✔ |  | Participants are Somali and Somali Bantu refugees. Even though all participants are refugees, their experiences might not be generalizable to refugees from other parts of the world. |
|  | Are measurements appropriate regarding both the outcome and intervention (or exposure)? | ✔ |  |  |  |
|  | Are there complete outcome data? | ✔ |  |  |  |
|  | Are the confounders accounted for in the design and analysis? | ✔ |  |  |  |
|  | During the study period, is the intervention administered (or exposure occurred) as intended? | ✔ |  |  |  |
| Quantitative non-randomized  Kira et al. (2015) | Are the participants representative of the target population? |  | ✔ |  | Participants are refugees, but they are also the children of Iraqi torture survivors. Therefore, their experiences and trauma might not be generalizable to the majority of refugee children. |
|  | Are measurements appropriate regarding both the outcome and intervention (or exposure)? | ✔ |  |  |  |
|  | Are there complete outcome data? | ✔ |  |  |  |
|  | Are the confounders accounted for in the design and analysis? | ✔ |  |  |  |
|  | During the study period, is the intervention administered (or exposure occurred) as intended? | ✔ |  |  |  |
| Mixed methods  Elswick et al. (2022) | Is there an adequate rationale for using a mixed methods design to address the research question? | ✔ |  |  |  |
|  | Are the different components of the study effectively integrated to answer the research question? | ✔ |  |  |  |
|  | Are the outputs of the integration of qualitative and quantitative components adequately interpreted? | ✔ |  |  |  |
|  | Are divergences and inconsistencies between quantitative and qualitative results adequately addressed? | ✔ |  |  |  |
|  | Do the different components of the study adhere to the quality criteria of each tradition of the methods involved? | ✔ |  |  | The quality of the qualitative and quantitative components of this study are high (see below). |
| Qualitative  Elswick et al. (2022) | Is the qualitative approach appropriate to answer the research question? | ✔ |  |  |  |
|  | Are the qualitative data collection methods adequate to address the research question? | ✔ |  |  |  |
|  | Are the findings adequately derived from the data? | ✔ |  |  |  |
|  | Is the interpretation of results sufficiently substantiated by data? | ✔ |  |  |  |
|  | Is there coherence between qualitative data sources, collection, analysis and interpretation? | ✔ |  |  |  |
| Quantitative non-randomized  Elswick et al. (2022) | Are the participants representative of the target population? |  | ✔ |  | The participants in this study are African refugee children (specific countries are unspecified). Even though all participants are refugees, their experiences might not be generalizable to refugees from other parts of the world. |
|  | Are measurements appropriate regarding both the outcome and intervention (or exposure)? | ✔ |  |  |  |
|  | Are there complete outcome data? | ✔ |  |  |  |
|  | Are the confounders accounted for in the design and analysis? | ✔ |  |  |  |
|  | During the study period, is the intervention administered (or exposure occurred) as intended? | ✔ |  |  |  |
| Mixed methods  Khawaja and Ramirez (2019) | Is there an adequate rationale for using a mixed methods design to address the research question? | ✔ |  |  |  |
|  | Are the different components of the study effectively integrated to answer the research question? | ✔ |  |  |  |
|  | Are the outputs of the integration of qualitative and quantitative components adequately interpreted? | ✔ |  |  |  |
|  | Are divergences and inconsistencies between quantitative and qualitative results adequately addressed? | ✔ |  |  |  |
|  | Do the different components of the study adhere to the quality criteria of each tradition of the methods involved? | ✔ |  |  | The quality of the qualitative and quantitative components of this study are high (see below). |
| Qualitative  Khawaja and Ramirez (2019) | Is the qualitative approach appropriate to answer the research question? | ✔ |  |  |  |
|  | Are the qualitative data collection methods adequate to address the research question? | ✔ |  |  |  |
|  | Are the findings adequately derived from the data? | ✔ |  |  |  |
|  | Is the interpretation of results sufficiently substantiated by data? | ✔ |  |  |  |
|  | Is there coherence between qualitative data sources, collection, analysis and interpretation? | ✔ |  |  |  |
| Quantitative non-randomized  Khawaja and Ramirez (2019) | Are the participants representative of the target population? |  | ✔ |  | The participants are a mixed migrant population. Even though nearly 50% are refugees, some of the participants are not refugees and, therefore, not representative of the target population. |
|  | Are measurements appropriate regarding both the outcome and intervention (or exposure)? | ✔ |  |  |  |
|  | Are there complete outcome data? | ✔ |  |  |  |
|  | Are the confounders accounted for in the design and analysis? | ✔ |  |  |  |
|  | During the study period, is the intervention administered (or exposure occurred) as intended? | ✔ |  |  |  |

| Study ID  **Table 3** Overview of Included Studies | Study location | Study setting | Study design | Sample characteristics | Country of origin and status | Sample size | Study aim(s) | Intervention | Acculturation element | Key points |
| --- | --- | --- | --- | --- | --- | --- | --- | --- | --- | --- |
| Beehler et al. (2012) | U.S.A. | School | Quantitative study; evaluation of intervention | 94 females and 55 males (mean age is 14.4 years old) | United States and Mexico (53.7%), Central America/Caribbean (14.8%), South America (13.4%), Africa (6%), South Asia (4.7%), Central/Eastern Europe (2.7%), Southeast Asia (2%), East Asia (1.3%), South Asia (1.3%); mixed migrant population (including refugees) | n = 149 | A within-subjects comparison of participants’ PTSD symptoms and functioning pre- and post-intervention | Cultural Adjustment and Trauma Services (CATS): a school-based intervention that uses adapted CBT, TF-CBT, relationship-building, and outreach services to support migrant youth | Acculturation informed the intervention’s school-based setting, comprehensive program, and flexible structure. Additionally, program staff created a “special acculturation group” to help participants at school. | Summary: Assess the effectiveness of CATS, an intervention using adapted CBT, TF-CBT, and relationship-building, and outreach services (adaptations include the use of bilingual clinicians, bicultural clinicians, clinicians with migrant backgrounds, culture brokers (members of migrant community groups), staff with experience in refugee resettlement, interpreters/translators (all services were available in the participants’ primary languages)) to improve PTSD symptoms and functioning of migrant youth.  Outcome measures: Child and Adolescent Functional Assessment Scale (CAFAS) and UCLA PTSD Reaction Index (PTSD-RI)  Results: Overall, there was a statistically significant improvement of participants’ functioning and reduction of PTSD symptoms. |
| Ellis et al. (2013) | U.S.A. | School | Quantitative study; evaluation of intervention | 11 females and 19 males (mean age is 13 years old) | Somalia (Somali and Somali Bantu refugees); all refugees | n = 30 | A within- subjects comparison of participants’ symptoms related to depression and PTSD, resource hardships, and treatment engagement pre- and post- intervention | Project Supporting the Health of Immigrant Families and Adolescents (Project SHIFA): a multi-tiered, school-based intervention culturally adapted to support refugee youth (tiers 1 and 2 involve group sessions of community and individual resilience building; tiers 3 and 4 involve more targeted and intensive therapy (trauma systems therapy based on trauma-focused cognitive behavioral principles) | Acculturation informed the services included in the intervention. For example, all participants joined a school-based language learning group that provided preventive skill building around managing acculturative stress and emotions. Researchers also collected data on acculturative hassles experienced by participants but found that the data did not affect the main outcomes of the study. | Summary: Assess the effectiveness of Project SHIFA, a school-based intervention involving culturally adapted CBT (adaptations include ensuring that 2 of the 4 clinicians delivering the intervention are Somali, having a Somali cultural broker contribute to treatment planning, having a Somali cultural broker present at participant interviews and home visits, acknowledging cultural approaches to healing, and linking the program to other refugee-related community services), on treatment engagement, PTSD and depression symptoms, and resource hardships.  Outcome measures: the Adolescent Post- War Adversities Scale (Somali version) (PWA), depression Self- Rating Scale (DSRS), UCLA PTSD Reaction Index for DSM–IV (Revision 1) (PTSD-RI), and War Trauma Screening Scale (WTSS)  Results: Overall, PTSD symptoms, depression symptoms and resource hardships decreased, and treatment engagement was high. However, improvements in resource hardships and mental health symptoms were not significantly different between participants receiving lower- intensity services and participants receiving higher-intensity services. |
| Elswick et al. (2022) | U.S.A. | After-school program | Mixed-methods (multiple time series research design); evaluation of intervention | 37 females and 51 males (12 to 18 years old) | Africa (unspecified); all refugees | n = 88 | A within-subjects comparison of participants’ trauma-related symptoms (including PTSD), effective coping skills, and functioning pre- and post-intervention. | Trauma Healing Club: an intervention that adapts and enhances Cognitive Behavioral Interventions for Trauma in Schools (CBITS) according to refugee families’ cultural backgrounds and needs | Literature on the effects of acculturation on African refugee families informed the researchers’ decision to create an intervention that targets trauma-related symptoms in school settings. | Summary: Assess the effectiveness of Trauma Healing Club, an after-school intervention incorporating adapted CBITS (adaptations include the addition of African drumming in each session to help participants process and regulate their emotions and pyramid mentoring, which involves training participants who complete the program so they can mentor and support future cohorts).  Outcome measures: the Child PTSD Symptom Scale (CPSS), Subjective Units of Distress Scale (SUDS), and Child Intervention Rating Profile (CIRP)  Results: PTSD symptoms decreased, effective coping skills increased, and functioning improved. |
| Khawaja and Ramirez (2019) | Australia | School and community | Mixed-methods; evaluation of intervention | 106 females and 120 males (mean age is 14 years old)—data missing for 3 participants | Asia (65%), Africa (12%), Middle East (7%), Oceania (6%), Europe (2%), South America (< 1%), data missing for 7%; nearly 50% are refugees | n = 229 | A within-subjects comparison of participants’ wellbeing and resilience related to acculturation processes pre- and post-intervention. | Building Resilience in Transcultural Australians (BRiTA Futures): an intervention that adapts CBT to build the resilience of youth experiencing acculturation related to migration and resettlement | The BRiTA Futures intervention’s emphasis on fostering positive relationships and support networks for participants is informed by literature on acculturation and the acculturative stress experienced by culturally and linguistically diverse youth. | Summary: Assess the effectiveness of BriTA Futures, an intervention adapting CBT principles (adaptations include use of bilingual staff as well as a focus on cross-cultural communication and cultural identity) to strengthen the resilience of culturally diverse youth experiencing acculturation related to migration and resettlement.  Outcome measures: a demographic form, an adapted version of the Multicultural Youth Resilience Questionnaire (MYRQ), General Health Questionnaire (GHQ), and review forms to collect comments and feedback from participants and facilitators  Results: Overall, participants’ wellbeing and resilience (related to acculturation) improved, and they learned new skills. However, factors such as being a refugee or a migrant, the format of the intervention, time spent in Australia, and gender did not have a statistically significant impacts on the improvements of wellbeing and resilience. |
| Kira et al. (2015) | U.S.A. | Summer day and after-school program | Quantitative | 14 females and 13 males (11 to 16 years old) | Iraq; all refugees | n = 27 | A within-subjects comparison of participants’ PTSD symptoms, complex PTSD symptoms, anxiety symptoms, depression symptoms, and global level of functioning pre- and post-intervention. | Multi-systemic, multi-component and multi-modal intervention model (MMMM): an intervention that adapts current, continuous, and cumulative trauma-focused cognitive behavioral therapy (CCC-TF-CBT) to support refugee youth | The MMMM intervention is informed by literature on acculturation/acculturative stress and aims to foster “healthy acculturation” for participants by working with participants at home/with their families, connecting participants with their peers through daily groups sessions, and utilizing community resources and services to help participants create meaningful connections with their new community. | Summary: Assess the effectiveness of MMMM, an intervention adapting CCC-TF-CBT (adaptations include the use of bilingual (Arabic) and bicultural (Middle Eastern/Iraqi) therapists, increased social support, and the addition of topics related to case management, legal aid, medical services, school, and community resources) and incorporating it into a program to support traumatized refugee children who are the children of torture survivors.  Outcome measures: Clinician Administered PTSD Scale (CAPS-2), Cumulative trauma disorder measure (CTD), the Center for Epidemiologic Studies Depression Measure (CES-D), Depression Anxiety Stress Scales-Anxiety (DASS-A), Global Assessment of Functioning (GAF)  Results: Overall, the participants’ PTSD, complex PTSD, depression, and anxiety symptoms decreased statistically significantly, and their functioning improved statistically significantly. |

Acculturation-informed CA-CBT. Beehler et al.’s intervention, Cultural Adjustment and Trauma Services (CATS), is a school-based intervention that utilizes adapted versions of CBT, trauma-focused-CBT, relationship-building resources, and outreach services to improve the mental health and wellbeing of migrant youth (Beehler et al., 2012). Ellis et al.’s intervention, Project Supporting the Health of Immigrant Families and Adolescents (Project SHIFA), is a multi-tiered, school-based intervention with components of group CBT and individual CBT that has been culturally adapted to support refugee youth (Ellis et al., 2013). Elswick et al.’s intervention is an after-school program, called “Trauma Healing Club,” that uses a version of Cognitive Behavioral Interventions for Trauma in Schools (CBITS) that has been adapted according to refugee families’ needs and cultural backgrounds (Elswick et al., 2022). Khawaja and Ramirez’s study investigates the effectiveness of Building Resilience in Transcultural Australians (BriTA) Futures, an intervention that uses acculturation-informed CA-CBT to build the resilience of youth with acculturative stress associated with migration and resettlement (Khawaja and Ramirez, 2019). Lastly, Kira et al.’s study explores the effectiveness of the Multi-systemic, multi-component and multi-modal intervention model (MMMM); an intervention based on an adapted version of current, continuous, and cumulative trauma-focused CBT that targets multiple traumas experienced by participants (CCC-TF-CBT) (Kira et al., 2015). For each of the interventions, researchers described their cultural adaptations. However, none of the studies demonstrated how researchers assessed the quality of the adaptations. See Table 3 for more information on the interventions and their cultural adaptations.

Each study’s intervention was informed by acculturation. Beehler et al.’s study highlights how the acculturation experienced by migrant youth informed their decision to make CATS a school-based intervention, have a flexible structure, be a comprehensive program (i.e. it offers several treatment options that address participants’ specific needs), and invite participants to join a “special acculturation group” at school (Beehler et al., 2012). In Ellis et al.’s study, acculturation informed the services included in Project SHIFA. All Somali students learning English were offered to join a school-based language learning group that provided preventive skill building around managing acculturative stress and emotions (Ellis et al., 2013). Researchers also collected data on acculturative hassles experienced by participants but found that the data did not affect the main outcomes of the study (Ellis et al., 2013). Elswick et al.’s study demonstrates how literature on the effects of acculturation on African refugee families informed their decision to design Trauma Healing Club to target trauma-related symptoms experienced by refugees and be delivered in school settings (Elswick et al., 2022). In Khawaja and Ramirez’s study,

| Study ID  **Table 4** CA-CBT for Refugee Youth and Families (Not Informed by Acculturation) | Study location | Study setting | Sample size | Sample characteristics | Country/region of origin | Intervention |
| --- | --- | --- | --- | --- | --- | --- |
| Doumit et al. (2020) | Lebanon | Community center | n = 31 | 16 females, 15 males (mean age: 14.2) | Syria | Creating Opportunities for Patient Empowerment (COPE) |
| Ehntholt et al. (2005) | United Kingdom | School | n = 26 | 9 females, 17 males (mean age: 13) | Kosovo, Sierra Leone, Turkey, Afghanistan, and Somalia | Group CBT |
| Gormez et al. (2017) | Turkey | School | n = 32 | 20 females, 12 males (age: 10-15) | Syria | Support group program based on CBT principles |
| King and Said (2019) | United Kingdom | Mental health clinic (NHS) | n = 14 | 1 female, 13 males (mean age: 14) | Afghanistan, Ethiopia, Somalia, and Sudan | Group CBT |
| O’Callaghan et al. (2015) | Democratic Republic of the Congo | School | n = 50 | 21 females, 29 males (age: 14-17) | Congolese | Group TF-CBT |
| Pfeiffer and Goldbeck (2017) | Germany | Clinical setting | n = 29 | 29 males (age: 14 to 18) | Afghanistan | Mein Weg (My Way) |
| Pfeiffer et al. (2018) | Germany | Clinical setting | n = 50 | 3 females, 47 males (mean age: 17) | Afghanistan, Syria, Gambia, Somalia, Iran, Eritrea, Senegal, Iraq, Ethiopia, Pakistan, Angola, Nigeria, Ivory Coast, Ghana, Guinea, Guinea-Bissau, and Kurdistan | Mein Weg (My Way) |
| Pfeiffer et al. (2019) | Germany | Clinical setting | n = 50 | 3 females, 47 males (mean age: 17) | Middle East and Africa (not specified) | Mein Weg (My Way) |
| Schottelkorb et al. (2012) | U.S.A | School | n = 31 | 14 females, 17 males (mean age: 9.16) | Burundi, Congo, Kenya, Liberia, Rwanda, Somalia, Tanzania, Myanmar, Nepal, Russia, Bosnia, Afghanistan, Iraq, Turkey, and Uzbekistan | CBT vs. child-centred play therapy |
| Sukale et al. (2020) | Germany | Clinical setting | n = 99 | 7 females, 92 males (age: 14-19) | Afghanistan, Syria, Gambia, Somalia, Iran, Eritrea, Senegal, Iraq, Ethiopia, Pakistan, Angola, Nigeria, Ivory Coast, Ghana, Guinea, Guinea-Bissau, and Kurdistan | Mein Weg (My Way) |
| Tsoupas (2011)—unpublished thesis written by Jenny Tsoupas at RMIT University | Australia | School | n = 21 | 9 females, 12 males (age: 7-12) | Mixed ethnicities (not specified) | The Rainbow Program |
| van Es et al. (2021) | Netherlands | Participant’s living location or another location of choice | n = 41 | 14 females, 27 males (mean age: 16.5) | Afghanistan, Eritrea, and Syria | Multimodal Trauma- Focused Treatment (CBT, eye movement desensitization and reprocessing (EMDR), and narrative exposure therapy) |
| Vickers (2005) | United Kingdom | Clinical setting | n = 2 | 1 female (14 years old) and 1 male (6 years old) | Africa and the Balkans | Individual CBT |

the researchers illustrate how BRiTA Futures’ emphasis on fostering youth’s positive relationships and support networks is informed by literature on acculturative stress experienced by culturally and linguistically diverse youth (Khawaja and Ramirez, 2019). Furthermore, in Kira et al.’s study, the researchers discuss how the MMMM’s emphasis on working with participants at home/with their families, connecting participants with peers through daily group sessions, and utilizing community resources and services to help participants create meaningful connections with their new community are informed by acculturation (Kira et al., 2015).

**Main Findings**

The studies included in this review used several outcome measures to assess participants’ mental health and wellbeing pre- and post-interventions and ultimately, evaluate the effectiveness of their interventions. Four studies assessed participants’ PTSD symptoms pre- and post-intervention using the Clinician Administered PTSD Scale (CAPS-2) (Kira et al.), the Child PTSD Symptom Scale (CPSS) (Elswick et al.) and the UCLA PTSD Reaction Index (PTSD-RI) (Beehler et al. and Ellis et al.) (Beehler et al., 2012; Ellis et al., 2013; Kira et al., 2015; Elswick et al., 2022). Three studies found that participants’ PTSD-related symptoms decreased significantly post-intervention (Beehler et al., 2012; Kira et al., 2015; Elswick et al., 2022). However, Ellis et al.’s study found that participants’ PTSD-related symptoms decreased but were not significantly different between receiving lower-intensity services and higher-intensity services. Two studies evaluated participants’ depression symptoms pre- and post-intervention using the Depression Self- Rating Scale (DSRS) and the Center for Epidemiologic Studies Depression Measure (CES-D) (Ellis et al., 2013; Kira et al., 2015). Both studies found a decrease in participants’ depression symptoms post-intervention, but Ellis et al. noticed that the decrease was not significantly different between lower-intensity and higher-intensity services (Ellis et al., 2013; Kira et al., 2015). Moreover, two studies evaluated participants’ overall functioning pre- and post-intervention using the Child and Adolescent Functional Assessment Scale (CAFAS) and Global Assessment of Functioning (GAF) (Beehler et al., 2012; Kira et al., 2015). Both studies found that participants’ functioning improved statistically significantly post-intervention.

The studies also used outcome measures to assess different areas of mental health and wellbeing. Ellis et al. measured participants’ resource hardships pre- and post-intervention using the Somali version of the Adolescent Post-War Adversities Scale (PWA) (Ellis et al., 2013). The researchers found that the participants’ resource hardships decreased over time, but the treatment tier participants were in did not have a significant impact on this decrease (Ellis et al., 2013). Ellis et al. also assessed treatment engagement by tracking how many participants remained in treatment until completion and found high levels of engagement (Ellis et al., 2013).

Kira et al.’s study also measured participants’ anxiety symptoms pre- and post-intervention using the Depression Anxiety Stress Scales-Anxiety (DASS-A). Kira et al. found a statistically significant decrease in participants’ anxiety symptoms post-intervention. Given that the MMMM intervention targets multiple traumas experienced by refugee youth, the researchers chose to use the Cumulative Trauma Disorder measure (CTD) to evaluate participants’ cumulative trauma in addition to PTSD symptoms (Kira et al., 2015). Kira et al. included this outcome measure (in addition to a PTSD outcome measure) because, given the complexity and multifaceted nature of refugee trauma, CTD measures are more adept at capturing participants’ different trauma profiles (Kira et al., 2015). Participants’ CTD-related symptoms decreased post-intervention (Kira et al., 2015).

Elswick et al.’s study also assessed participants’ ability to regulate feelings and emotions using the Subjective Units of Distress Scale (SUDS) pre- and post-intervention. Elswick et al. found that participants had higher SUDS scores at the beginning of the intervention and that these scores decreased over time (Elswick et al., 2022). Elswick et al.’s study also used the Child Intervention Rating Profile (CIRP) to investigate participants’ perceptions of the Trauma Healing Club (Elswick et al., 2022). At the beginning of the intervention, all participants thought it could be helpful but might negatively impact the way teachers and peers perceived them (Elswick et al., 2022). However, during the post-intervention assessment, all participants indicated that they thought the intervention was very helpful and their peers and teachers were very supportive (Elswick et al., 2022).

Khawaja and Ramirez’s study focussed on evaluating participants’ resilience using the Multicultural Youth Resilience Questionnaire (MYRQ), and wellbeing using the General Health Questionnaire (GHQ). The researchers found that participants’ resilience—in the form of positive coping strategies, optimism, engagement, coping ability, and personal/cultural strengths measured by responses to the MYRQ—significantly improved post-intervention (Khawaja and Ramirez, 2019). Additionally, researchers found that the participants’ psychological health and wellbeing also improved significantly (Khawaja and Ramirez, 2019).

Two studies also included qualitative analyses (Khawaja and Ramirez, 2019; Elswick et al., 2022). Elswick et al. included feedback from participants in their study. Participants mentioned that Trauma Healing Club made them feel happier, more relaxed, less stressed (one person said the club’s drumming activity helped with this), helped them make new friends, and was fun (Elswick et al., 2022). Moreover, Khawaja and Ramirez included feedback and comments collected from participants in the study. Participants mentioned that they were satisfied with the BRiTA Futures program, enjoyed learning about the impact of culture, developed new conflict-management, problem-solving, and communication skills, felt more self-confident, happier, more positive, more able to manage their emotions, and valued themselves more (Khawaja and Ramirez, 2019).

Even though acculturation informed the interventions in each of these studies, only two of the studies used acculturation-related measures. Ellis et al. collected data on participants’ acculturative hassles using the Acculturative Hassles Inventory (Ellis et al., 2013). However, Ellis et al. neither included nor discussed the data on acculturative hassles in their article because the changes in the measure pre- and post-intervention were not found to be associated with the main outcomes of the study (Ellis et al., 2013). However, a part of Project SHIFA (referred to as Tier 2) was heavily informed by acculturation. Tier 2 was delivered by two Somali and two non-Somali clinicians and focused on preventive skill building around managing acculturative stress and emotions (Ellis et al., 2013). All participants participated in Tier 2, but only half of these participants were identified as being especially at-risk and asked to partake in additional intensive parts of the program (Tiers 3 and 4) (Ellis et al., 2013). Ellis et al. mention that the PTSD symptoms and resource hardships of all participants improved, regardless of their tier levels (Ellis et al., 2013). Given that all participants took part in Tier 2 (which involved building skills to help manage acculturative stress and emotions), working on refugee youth’s acculturation-related issues might contribute to improving their PTSD symptoms and resource hardships (Ellis et al., 2013). Nonetheless, this is speculative due to the lack of data on acculturation included in Ellis et al.’s study (Ellis et al., 2013). Secondly, Khawaja and Ramirez measured youth’s perceived acculturation-related resilience using an adapted version of the MYRQ (Khawaja and Ramirez, 2019). Khawaja and Ramirez’s findings indicate that their intervention was associated with an increase in participants acculturation-related resilience (Khawaja and Ramirez, 2019). Khawaja and Ramirez assert that more research is required to fully establish the MYRQ’s validity (Khawaja and Ramirez, 2019). However, the study still highlights how an acculturation-informed CA-CBT intervention that emphasizes fostering youth’s relationships and support networks, can build youth’s acculturation-related resilience and positively impact their acculturation process.

Despite not using acculturation-related measures in their studies, Beehler et al., Elswick et al., and Kira et al. demonstrate how acculturation informed their interventions. Beehler et al. found that school settings are where children’s acculturative and adjustment challenges largely transpire (Beehler et al., 2012). Consequently, research on acculturation informed Beehler et al.’s decision to make CATS school-based as well as provide participants with special acculturation groups to help them adjust to their new environment (Beehler et al., 2012). Beehler et al. also mention that many participants in the CATS intervention reportedly experienced acculturation-related stress and disruptions during treatment (Beehler et al., 2012). Even though CATS was informed by acculturation and participants’ acculturative stress, Beehler et al. did not officially measure acculturation in their study (Beehler et al., 2012). Furthermore, Elswick et al. discuss research on the acculturation-related challenges of African refugee families and designed Trauma Healing Club accordingly (Elswick et al., 2022). Elswick et al. assert that the combination of traumatic experiences and acculturation-related challenges faced by African refugees predisposes them to developing PTSD symptoms, other trauma symptoms, and additional mental health issues (Elswick et al., 2022). Therefore, to effectively tackle the acculturation-related challenges as well as the trauma experienced by African refugees, Elswick et al. made sure Trauma Healing Club used cultural support and resources so that it could address participants’ acculturation-related needs (Elswick et al., 2022). Even though Elswick et al. took acculturation into account when creating Trauma Healing Club, they did not measure acculturation in any way (Elswick et al., 2022). Lastly, Kira et al. assert that most interventions for refugee youth typically focus on single past traumas and rarely address additional present stressors and challenges, like those related to acculturation (Kira et al., 2015). As a result, Kira et al. developed the MMMM so that refugee youth could receive an intervention that addresses their multiple traumas and additional stressors including acculturation-related difficulties (Kira et al., 2015). To help participants with acculturation-related and other challenges, the MMMM intervention aims to work with participants at home/with their families, connect participants with their peers, and utilize community resources and services to help participants create meaningful connections (Kira et al., 2015). However, even though the MMMM intervention was informed by acculturation and the researchers mention that participants experienced acculturative stress, Kira et al. did not formally assess acculturation in their study (Kira et al., 2015). Therefore, even though Beehler et al., Elswick et al., and Kira et al. describe how their interventions have been informed by acculturation and acknowledge that acculturation could have contributed to the participants’ experiences and outcomes, they have not formally measured acculturation or used any acculturation-related assessments in their studies. Lastly, even though the researchers for each of the included studies explained how their CA-CBT interventions were informed by acculturation, there were very few formal measures of acculturation. The lack of formal measurements of acculturation makes it difficult to understand how acculturation specifically contributed to participants’ outcomes.

**Discussion**

The goal of this systematic review is to explore the effectiveness of acculturation-informed CA-CBT in reducing trauma-related symptoms such as PTSD, anxiety, or depression as well as improving day-to-day functioning, effective coping skills, and resilience among refugee youth and family populations. Each of the five studies included in this review found improvements for the participants receiving their interventions. Cultural adaptations made to the CBT interventions included the use of translators, interpreters, bilingual clinicians, bicultural clinicians, clinicians with migrant backgrounds, staff with experience in refugee resettlement, members of local migrant community groups, acknowledging and working with cultural approaches to healing, and including culturally-relevant activities in the program among other things (Beehler et al., 2012; Ellis et al., 2013; Kira et al., 2015; Khawaja and Ramirez, 2019; Elswick et al., 2022). Acculturation was accounted for in a variety of ways. The interventions included preventive skill building around managing acculturative stress, helped strengthen relationships and support networks to build resilience and positively impact the acculturation process, provided special school-based acculturation groups, used culturally relevant support and resources to address acculturation-related needs, and worked with participants’ families and connected them with peers in their community to help with acculturation-related challenges (Beehler et al., 2012; Ellis et al., 2013; Kira et al., 2015; Khawaja and Ramirez, 2019; Elswick et al., 2022). Two studies used acculturation-related measures (Ellis et al., 2013; Khawaja and Ramirez, 2019); Ellis et al. found that the data they collected on participants’ acculturative hassles were not associated with the main outcomes of their study, however, Khawaja and Ramirez found that their intervention was associated with an improvement in participants’ acculturation-related resilience. Due to the lack of formal assessments of acculturation, conclusions are difficult to draw. Nonetheless, all of the interventions demonstrated mental health and wellbeing improvements. Therefore, this review presents support for the use of acculturation-informed CA-CBT interventions when working with refugee youth.

     	The conclusions of this review are similar to those of other related systematic reviews (Tyrer and Fazel, 2014; Lawton and Spencer, 2021; Taylor et al., 2022). However, to the researchers’ knowledge, this is the first systematic review to focus on acculturation-informed CA-CBT interventions for refugee youth and families. Consequently, this review was able to contribute evidence for both CA-CBT interventions and acculturation-informed CA-CBT interventions. Nevertheless, the conclusions of this review should be taken with caution due to the limited statistical analysis of the studies’ findings and the fact that they might not meet rigorous research standards (e.g. the use of random selection, randomization, blinding methods, or control groups).

**Limitations**

There are several limitations concerning the studies included in this review as well as this review as a whole. Firstly, the small sample sizes and heterogeneity of participants might make the findings of these studies less generalizable (Ellis et al., 2013; Kira et al., 2015; Elswick et al., 2022). The participants in three studies were all refugees (Ellis et al., 2013; Kira et al., 2015; Elswick et al., 2022). However, each of these studies focused on refugees from specific ethnic or regional origins. Even though all the participants in these three studies are refugees, their experiences might not be generalizable to refugees of other demographics and ethnic groups. Moreover, the participants in two studies are of mixed migrant populations (Beehler et al., 2012; Khawaja and Ramirez, 2019). Although these participant groups include refugee youth, the experiences of other migrant youth might not be generalizable to refugees.

There is also a notable lack of comparison or control groups within these studies. The interventions explored were administered over 12 weeks (Elswick et al.’s study), a year (Kira et al. and Ellis et al.’s studies), 3 years (Beehler et al.’s study), and 6 years (Khawaja and Ramirez’s study). The lack of control or comparison groups here makes it challenging to draw conclusions about whether the interventions were specifically responsible for participants’ improvements or if the participants improved over time due to other reasons. Other variables like differences in training and experience of those administering the interventions, and differences in participants’ English and language skills were also not controlled, compared, or measured. Three studies had translators or bilingual clinicians, and Khawaja and Ramirez also suggested incorporating picture-based scales to help participants overcome language barriers (Beehler et al., 2012; Kira et al., 2015; Khawaja and Ramirez, 2019). Therefore, as some researchers mention, there are several factors that could have influenced participant outcomes that were not formally measured (Kira et al., 2015). Moreover, the outcomes for parents, teachers, and other individuals indirectly involved in the interventions were not assessed.

The ways acculturation was used to inform interventions differed across studies. This makes it difficult to compare the types of interventions included in this review. Additionally, the researchers did not explain how specific components of their interventions contributed to participants’ outcomes. Consequently, it is difficult to understand which aspects of the acculturation-informed CA-CBT contributed to participants’ improvements. Most importantly, none of the studies were compared to interventions that were not acculturation-informed or culturally adapted. Kira et al. mention that the Iraqi refugee youth participants were all unresponsive to traditional TF-CBT and psychiatric interventions but showed improvements after receiving the MMMM intervention (Kira et al., 2015). However, formal comparisons were not included in any of the studies. This severely limits this review’s ability to ascertain if acculturation-informed CA-CBT provides notable benefits for refugee youth and families when compared to CA-CBT interventions that are not informed by acculturation or treatment as usual (CBT and TF-CBT).

This review has illustrated there is a scarcity of research on acculturation-informed CA-CBT for refugee youth and families. An additional limitation for this review is that the study selection process was conducted between April 14, 2022, and June 14, 2022 and completed before studies from 2023, 2024, and 2025 became available. As a result, the timeline of this systematic review limited the inclusion of any literature post-2022. Even though the literature existing at the time of this review still provides relevant insight into the effectiveness of acculturation-informed CA-CBT interventions for refugee youth and families, it would be wise to conduct similar systematic reviews now that researchers have access to more studies to include findings from more recent studies as well. Alto et al.’s practice paper outlines a program design for an intervention that is informed by acculturation and incorporates elements of CA-CBT (Alto et al., 2021). Alto et al.’s program is informed by acculturation in several ways, most notably by dedicating an entire program session to acculturation and identity to help participants’ work on related challenges (Alto et al., 2021). Although Alto et al.’s paper was excluded from this review because it is not a formal study, the researchers highlighted several relevant recommendations for interventions (building social relationships, finding ways to positively impact acculturation, and discussing intercultural competences, etc.) (Alto et al., 2021). Additionally, Rosner et al.’s study protocol is for Better Care, an acculturation-informed CA-CBT intervention that incorporates Pfeiffer et al.’s (2017, 2018, and 2019) Mein Weg intervention (mentioned in Table 4) (Rosner et al., 2020; Pfeiffer and Goldbeck, 2017; Pfeiffer et al., 2018; Pfeiffer et al., 2019). At the time of this review, the results of Rosner et al.’s study had not been published. Nonetheless, Alto et al.’s practice paper and Rosner et al.’s protocol indicate that more studies on acculturation-informed CA-CBT interventions for refugee youth are in the works and will contribute to the growing evidence base.

Another limitation is that, given how cultural sensitivity is a core aspect of TF-CBT, TF-CBT might be considered as inherently qualifying as CA-CBT under the authors’ definition. TF-CBT is considered culturally responsive due to its flexibility, collaborative nature, and inclusion of family and caregivers (Cohen et al., 2018; Deblinger et al., 2016). This can help with ensuring the treatment process is in accord with the client’s principles, beliefs, cultural norms, language, etc. (Cohen et al., 2018; Deblinger et al., 2016). Therefore, it could be argued that TF-CBT falls under the authors' description of CA-CBT. However, this review aimed to examine CBT interventions that included specific cultural adaptations to treatment, taking into account factors such as the client’s cultural background, values, and language (Bernal et al., 2009; Hinton et al., 2012; Taylor et al., 2022). Therefore, while TF-CBT is inherently culturally responsive and flexible, only studies that included TF-CBT interventions with specific cultural adaptations were considered to qualify as CA-CBT interventions in this review. That being said, given the substantial overlap between CA-CBT and TF-CBT, it would be beneficial for future reviews and analyses to broaden the scope to acculturation-informed TF-CBT studies. Additionally, the included studies did not compare acculturation-informed CA-CBT with CA-CBT, traditional CBT, or TF-CBT not informed by acculturation. Therefore, it would also be worthwhile for future reviews and analyses to compare the acculturation-informed CA-CBT studies included in this review with the CA-CBT interventions displayed in Table 4.

Furthermore, even though grey literature was searched, this review is still at-risk for publication bias because other unpublished sources could have been missed. Given that this review only included studies written (or translated into) English, this review runs the risk of excluding potentially pertinent sources in other languages.

**Research and Clinical Implications**

This review highlights the lack of available studies on acculturation-informed CA-CBT interventions for refugee youth. The five interventions included in this review are promising examples of how acculturation-informed CA-CBT interventions can address and improve refugee youth’s mental health and wellbeing. The studies found that refugee youth’s symptoms related to trauma, PTSD, anxiety, and depression as well as functioning, effective coping skills, resource hardships, treatment engagement, and resilience associated with acculturation all improved (Beehler et al., 2012; Ellis et al., 2013; Kira et al., 2015; Khawaja and Ramirez, 2019; Elswick et al., 2022). However, despite promising results, more studies need to be conducted and reviewed to allow for a stronger understanding of how acculturation-informed CA-CBT interventions can support refugee youth. Future studies should aim to be more methodologically rigorous by increasing sample sizes, including comparison or control groups, including measures for relevant variables like acculturation, and assessing the efficacy of intervention adaptation. Lastly, it might be useful for future reviews and analyses to compare the acculturation-informed CA-CBT studies included here with the CA-CBT interventions displayed in Table 4.

These studies also have clinical implications. As highlighted in Table 3, the included studies provide concrete examples of how the interventions were culturally adapted and informed by acculturation. These include flexibility in interventions, the creation of a specific acculturation group for students, and activities to build skills for managing acculturative stress and emotions (Beehler et al., 2012; Ellis et al., 2013). Other examples include delivering interventions in school settings, finding ways of also working with participants and their families at home, helping youth form positive peer relationships and expand their support networks, as well as utilizing community resources and services to strengthen community ties (Beehler et al., 2012; Ellis et al., 2013; Elswick et al., 2022; Khawaja and Ramirez, 2019; Kira et al., 2015).

Specifically in terms of treatment delivery context, all studies were implemented and delivered in school or after-school settings. Researchers assert working in school settings reduced barriers to treatment for refugee families, provided children with a familiar physical space for therapy, and opened the possibility for other professionals (teachers, nurses, other school staff, etc.) who receive proper training to help (Ellis et al., 2013; Tyrer and Fazel, 2014; Khawaja and Ramirez, 2019). Researchers have also found that children’s acculturative and adjustment challenges largely occur in school settings (Beehler et al., 2012). Therefore, interventions for refugee youth informed by acculturation might benefit from being administered to children in schools.

Moreover, researchers have found that programs promoting family involvement, addressing cultural ties, including activities related to participants’ cultural heritage, and fostering connections with peers and the community can help develop protective factors, making them more resilient to the trauma-related symptoms they experience (Ellis et al., 2013; Elswick et al., 2022). Further research is needed to understand how these specific adaptations promote these protective factors. What is more, researchers aimed to make their interventions more accessible to refugee youth by including practitioners who either had migrant backgrounds, were bicultural, or shared the participants’ ethnic background (Beehler et al., 2012; Ellis et al., 2013; Kira et al., 2015). To make this more feasible, researchers argue that efforts should be made to build accessible gateways to mental health professions in underrepresented communities (Ellis et al., 2013).

**Conclusion**

Even though acculturation-informed CA-CBT interventions for refugee youth and families are not well established, there is growing evidence that supports the use of these interventions. Ultimately, this systematic review demonstrates how five acculturation-informed CA-CBT interventions have been designed and implemented to meet the unique needs and address the complex challenges experienced by refugee youth. This systematic review provides information for clinicians, mental health professionals, researchers, public health professionals, policymakers, and individuals working for organizations that assist migrant youth. Further evaluation of acculturation-informed CA-CBT interventions is needed before concrete conclusions can be drawn and interventions are disseminated. Yet, despite there being several limitations to this review and its included studies, its findings indicate acculturation-informed CA-CBT interventions show promising outcomes for refugee youth. Therefore, to understand how mental health professionals can best support refugee youth and families during the resettlement process, further rigorous research on acculturation-informed CA-CBT is needed.

**References**

Alto, M. E., Bantelman, E., Manly, J. T., Hathaway, A., Knight, S., Frounfelker, R. L., & Petrenko, C. (2021). The Development of a Mental Health Program for Unaccompanied Minors in the United States*.* *International Journal for the Advancement of Counselling*, *44*(1), 164–196. <https://doi.org/10.1007/s10447-021-09442-0>

American Psychological Association. (2017). *What is Cognitive Behavioral Therapy*? PTSD Clinical Practice Guideline. <https://www.apa.org/ptsd-guideline/patients-and-families/cognitive-behavioral.pdf>

Barrett, P. M., Sonderegger, R., & Xenos, S. (2003). Using Friends to Combat Anxiety and Adjustment Problems among Young Migrants to Australia: A National Trial. *Clinical Child Psychology and Psychiatry*, *8*(2), 241–260. <https://doi.org/10.1177/1359104503008002008>

Bean, Derluyn, I., Eurelings-Bontekoe, E., Broekaert, E., & Spinhoven, P. (2007). Comparing Psychological Distress, Traumatic Stress Reactions, and Experiences of Unaccompanied Refugee Minors With Experiences of Adolescents Accompanied by Parents. *The Journal of Nervous and Mental Disease*, *195*(4), 288–297. <https://doi.org/10.1097/01.nmd.0000243751.49499.93>

Beehler, S., Birman, D., & Campbell, R. (2012). The Effectiveness of Cultural Adjustment and Trauma Services (CATS): Generating Practice-Based Evidence on a Comprehensive, School-Based Mental Health Intervention for Immigrant Youth. *American Journal of Community Psychology*, *50*(1-2), 155–168. <https://doi.org/10.1007/s10464-011-9486-2>

Bernal, Jiménez-Chafey, M. I., & Domenech Rodríguez, M. M. (2009). Cultural Adaptation of Treatments: A Resource for Considering Culture in Evidence-Based Practice. *Professional Psychology, Research and Practice*, *40*(4), 361–368. <https://doi.org/10.1037/a0016401>

Berry, J. W. (1997). Immigration, acculturation, and adaptation. *Applied Psychology: An International Review,* *46*(1), 5–34. <https://doi.org/10.1080/026999497378467>

Buchanan, Z. E., Abu-Rayya, H. M., Kashima, E., & Paxton, S. J. (2017). The Interconnection between Acculturation and Subjective and Social Wellbeing among Refugee Youth in Australia. *Journal of Refugee Studies*, *30*(4), 511–529. <https://doi.org/10.1093/jrs/few040>

Cohen, J. A., Deblinger, E., & Mannarino, A. P. (2018). Trauma-focused cognitive behavioral therapy for children and families. Psychotherapy Research, 28(1), 47–57. https://doi.org/10.1080/10503307.2016.1208375

Deblinger, E., Pollio, E., & Dorsey, S. (2016). Applying Trauma-Focused Cognitive–Behavioral Therapy in Group Format. *Child Maltreatment*, *21*(1), 59–73. https://doi.org/10.1177/1077559515620668

Davidson, G. R., Murray, K. E., & Schweitzer, R. D. (2010). Review of Refugee Mental Health Assessment: Best Practices and Recommendations. *Journal of Pacific Rim Psychology*, *4*(1), 72–85. <https://doi.org/10.1375/prp.4.1.72>

Doumit, Kazandjian, C., & Militello, L. K. (2020). COPE for Adolescent Syrian Refugees in Lebanon: A Brief Cognitive–Behavioral Skill-Building Intervention to Improve Quality of Life and Promote Positive Mental Health. *Clinical Nursing Research*, *29*(4), 226–234. <https://doi.org/10.1177/1054773818808114>

Ehntholt, Smith, P. A., & Yule, W. (2005). School-based Cognitive-Behavioural Therapy Group Intervention for Refugee Children who have Experienced War-related Trauma. *Clinical Child Psychology and Psychiatry*, *10*(2), 235–250. <https://doi.org/10.1177/1359104505051214>

Ellis, B. H., Miller, A. B., Abdi, S., Barrett, C., Blood, E. A., & Betancourt, T. S. (2013). Multi-Tier Mental Health Program for Refugee Youth. *Journal of Consulting and Clinical Psychology*, *81*(1), 129–140. <https://doi.org/10.1037/a0029844>

Elswick, S., Washington, G., Mangrum-Apple, H., Peterson, C., Barnes, E., Pirkey, P., & Watson, J. (2022). Trauma Healing Club: Utilizing Culturally Responsive Processes in the Implementation of an After-School Group Intervention to Address Trauma Among African Refugees. *Journal of Child & Adolescent Trauma*, *15*(1), 155–166. <https://doi.org/10.1007/s40653-021-00387-5>

Genç, E. (2022). The effectiveness of trauma treatment approaches in refugee children and adolescents: A systematic review*. Children and Youth Services Review*, *141*, 106602. <https://doi.org/10.1016/j.childyouth.2022.106602>

Gormez, Kılıç, H. N., Orengul, A. C., Demir, M. N., Mert, E. B., Makhlouta, B., Kınık, K., & Semerci, B. (2017). Evaluation of a school-based, teacher-delivered psychological intervention group program for trauma-affected Syrian refugee children in Istanbul, Turkey. *Klinik Psikofarmakoloji Bülteni*, *27*(2), 125–131. <https://doi.org/10.1080/24750573.2017.1304748>

Griner, D., & Smith, T. B. (2006). Culturally adapted mental health intervention: A meta-analytic review. *Psychotherapy (Chicago, Ill.)*, *43*(4), 531–548. <https://doi.org/10.1037/0033-3204.43.4.531>

Halasa, Hamdan-Mansour, A. M., Salami, I., & Alenezi, A. (2020). Post-Traumatic Stress and Social Anxiety Among Children of Syrian Refugees in Jordan. *International Journal of Mental Health and Addiction*, *18*(6), 1611–1619. <https://doi.org/10.1007/s11469-020-00250-y>

Hinton, D. E., Rivera, E. I., Hofmann, S. G., Barlow, D. H., & Otto, M. W. (2012). Adapting CBT for traumatized refugees and ethnic minority patients: Examples from culturally adapted CBT (CA-CBT). *Transcultural Psychiatry*, *49*(2), 340–365. <https://doi.org/10.1177/1363461512441595>

Hong, Q.N., Pluye, P., Fàbregues, S., Bartlett, G., Boardman, F., Cargo, M., et al. (2018). *Mixed Methods Appraisal Tool (MMAT), version 2018*. Registration of Copyright (#1148552), Canadian Intellectual Property Office, Industry Canada. <https://doi.org/10.3233/EFI-180221>

Huey, & Polo, A. J. (2008). Evidence-Based Psychosocial Treatments for Ethnic Minority Youth. *Journal of Clinical Child and Adolescent Psychology*, *37*(1), 262–301. <https://doi.org/10.1080/15374410701820174>

Keles, S., Friborg, O., Idsøe, T., Sirin, S., & Oppedal, B. (2018). Resilience and acculturation among unaccompanied refugee minors. *International Journal of Behavioral Development*, *42*(1), 52–63. <https://doi.org/10.1177/0165025416658136>

Khawaja, N. G., & Ramirez, E. (2019). Building resilience in transcultural adolescents: An evaluation of a group program. *Journal of Child and Family Studies*, *28*(11), 2977-2987. doi:https://doi.org/10.1007/s10826-019-01473-x

King, & Said, G. (2019). Working with unaccompanied asylum-seeking young people: Cultural considerations and acceptability of a cognitive behavioural group approach. *Cognitive Behaviour Therapist*, *12*, 1-23. <https://doi.org/10.1017/S1754470X18000260>

Kira, I. A., Lewandowski, L., Ashby, J. S., & Omidy, A. Z. (2015). Development and Piloting the MMMM Ecological Interactive Open Systems Model: A Prospective One-Year Treatment Outcome of Children of Tortured Refugees. *Psychology*, *6*, 1277-1296. <http://dx.doi.org/10.4236/psych.2015.610125>

Lawson, & Quinn, J. (2013). Complex Trauma in Children and Adolescents: Evidence-Based Practice in Clinical Settings. *Journal of Clinical Psychology*, *69*(5), 497–509. <https://doi.org/10.1002/jclp.21990>

Lawton, K., & Spencer, A. (2021). A Full Systematic Review on the Effects of Cognitive Behavioural Therapy for Mental Health Symptoms in Child Refugees. *Journal of Immigrant and Minority Health*, *23*(3), 624–639. <https://doi.org/10.1007/s10903-021-01151-5>

Mengistu, B.S., Manolova, G. (2019). Acculturation and mental health among adult forced migrants: a meta-narrative systematic review protocol. *Systematic Reviews* 8, 184 <https://doi.org/10.1186/s13643-019-1103-8>

Moyes, Nutman, G., & Mirman, J. H. (2022). The Efficacy of Targeted Mindfulness-Based Interventions for Improving Mental Health and Cognition Among Youth and Adults with ACE Histories: A Systematic Mixed Studies Review. *Journal of Child & Adolescent Trauma*. <https://doi.org/10.1007/s40653-022-00454-5>

Naeem, F. (2019). Cultural adaptations of CBT: A summary and discussion of the Special Issue on Cultural Adaptation of CBT. *Cognitive Behaviour Therapist*, *12*. <https://doi.org/10.1017/S1754470X19000278>

Nasıroğlu, Çeri, V., Erkorkmaz, Ü., & Semerci, B. (2018). Determinants of psychiatric disorders in children refugees in Turkey’s Yazidi refugee camp. *Psychiatry and Clinical Psychopharmacology*, *28*(3), 291–299. <https://doi.org/10.1080/24750573.2017.1422958>

Nocon, A., Eberle-Sejari, R., Unterhitzenberger, J., & Rosner, R. (2017). The effectiveness of psychosocial interventions in war-traumatized refugee and internally displaced minors: systematic review and meta-analysis. *European Journal of Psychotraumatology*, *8*(sup2), 1388709. <https://doi.org/10.1080/20008198.2017.1388709>

O’Callaghan, McMullen, J., Shannon, C., & Rafferty, H. (2015). Comparing a trauma focused and non trauma focused intervention with war affected Congolese youth: a preliminary randomised trial. *Intervention (Amstelveen, Netherlands)*, *13*(1), 28–44. <https://doi.org/10.1097/WTF.0000000000000054>

Oppedal, B. & Idsoe, T. (2015). The role of social support in the acculturation and mental health of unaccompanied minor asylum seekers. *Scandinavian Journal of Psychology*, *56*, 203–211. <https://doi.org/10.1111/sjop.12194>

Pacione, Measham, T., & Rousseau, C. (2013). Refugee Children: Mental Health and Effective Interventions. *Current Psychiatry Reports*, *15*(2), 341–349. <https://doi.org/10.1007/s11920-012-0341-4>

Page, M. J., McKenzie, J. E., Bossuyt, P. M., Boutron, I., Hoffmann, T. C., Mulrow, C. D., Shamseer, L., Tetzlaff, J. M., Akl, E. A., Brennan, S. E., Chou, R., Glanville, J., Grimshaw, J. M., Hróbjartsson, A., Lalu, M. M., Li, T., Loder, E. W., Mayo-Wilson, E. McDonald, S.,… Moher, D. (2021). The PRISMA 2020 statement: an updated guideline for reporting systematic reviews. *BMJ (Online)*, *372*, n71–n71. <https://doi.org/10.1136/bmj.n71>

Pfeiffer, & Goldbeck, L. (2017). Evaluation of a Trauma‐Focused Group Intervention for Unaccompanied Young Refugees: A Pilot Study. *Journal of Traumatic Stress*, *30*(5), 531–536. <https://doi.org/10.1002/jts.22218>

Pfeiffer, Sachser, C., Rohlmann, F., & Goldbeck, L. (2018). Effectiveness of a trauma‐focused group intervention for young refugees: a randomized controlled trial. *Journal of Child Psychology and Psychiatry*, *59*(11), 1171–1179. <https://doi.org/10.1111/jcpp.12908>

Pfeiffer, E., Sachser, C., Tutus, D., Fegert, J. M., & Plener, P. L. (2019). Trauma-focused group intervention for unaccompanied young refugees: "Mein Weg"-predictors of treatment outcomes and sustainability of treatment effects. *Child and Adolescent Psychiatry and Mental Health*, *13*, 18. <https://doi.org/10.1186/s13034-019-0277-0>

Rosner, R., Sachser, C., Hornfeck, F., Kilian, R., Kindler, H., Muche, R., Müller, L. R. F., Thielmann, J., Waldmann, T., Ziegenhain, U., Unterhitzenberger, J., & Pfeiffer, E. (2020). Improving mental health care for unaccompanied young refugees through a stepped-care approach versus usual care+: study protocol of a cluster randomized controlled hybrid effectiveness implementation trial. *Trials*, *21*(1), 1013–1013. <https://doi.org/10.1186/s13063-020-04922-x>

Schottelkorb, Doumas, D. M., & Garcia, R. (2012). Treatment for childhood refugee trauma: A randomized, controlled trial. *International Journal of Play Therapy*, *21*(2), 57–73. <https://doi.org/10.1037/a0027430>

Sukale, T., Rassenhofer, M., Kirsch, V., & Pfeiffer, E. (2020). Niedrigschwellige traumafokussierte Gruppeninterventionen für traumatisierte Jugendliche mit Fluchterfahrung: Der Einfluss von nicht-suizidalem selbstverletzendem Verhalten und Suizidalität. Zeitschrift Für Psychiatrie*, Psychologie Und Psychotherapie*, *68*(1), 52–63. <https://doi.org/10.1024/1661-4747/a000404>

Tan, Y.W., Burgess, G. H., & Green, R. J. (2020). The effects of acculturation on neuropsychological test performance: A systematic literature review. *Clinical Neuropsychologist*, *35*(3), 541–571. <https://doi.org/10.1080/13854046.2020.1714740>

Taylor, A., Radford, G., & Calia, C. (2022). Review: Cultural adaptations to psychosocial interventions for families with refugee/asylum-seeker status in the United Kingdom – a systematic review. *Child and Adolescent Mental Health*. <https://doi.org/10.1111/camh.12547>

Titzmann, P. F., & Lee, R. M. (2022). New temporal concepts of acculturation in immigrant youth. *Child Development Perspectives*, *16*, 165–172. <https://doi.org/10.1111/cdep.12458>

Troup, Fuhr, D. C., Woodward, A., Sondorp, E., & Roberts, B. (2021). Barriers and facilitators for scaling up mental health and psychosocial support interventions in low- and middle-income countries for populations affected by humanitarian crises: a systematic review. *International Journal of Mental Health Systems*, *15*(1), 5–5. <https://doi.org/10.1186/s13033-020-00431-1>

Tyrer, R. A., & Fazel, M. (2014). School and community-based interventions for refugee and asylum seeking children: a systematic review. *PloS One*, *9*(5), e89359. <https://doi.org/10.1371/journal.pone.0089359>

UN General Assembly. (1951, July). *Convention relating to the Status of Refugees*. United Nations, Treaty Series, vol. 189, p. 137. <https://www.refworld.org/docid/3be01b964.html>

UNHCR (United Nations High Commissioner for Refugees). (2023a, October 25). *UNHCR: Forced displacement continues to grow as conflicts escalate*. UNHCR: The UN Refugee Agency. <https://www.unhcr.org/uk/news/press-releases/unhcr-forced-displacement-continues-grow-conflicts-escalate>

UNHCR (United Nations High Commissioner for Refugees). (2023b, October 25). *Mid-Year Trends 2023*. UNHCR: The UN Refugee Agency. <https://www.unhcr.org/uk/mid-year-trends>

UNICEF. (2022, June). *Child displacement*. UNICEF Data - Child Statistics. <https://data.unicef.org/topic/child-migration-and-displacement/displacement/>

Unterhitzenberger, J., & Rosner, R. (2016). Case report: manualized trauma-focused cognitive behavioral therapy with an unaccompanied refugee minor girl. *European Journal of Psychotraumatology, 7*, 29246. <https://doi.org/10.3402/ejpt.v7.29246>

van Es, C. M., Sleijpen, M., Velu, M. E., Boelen, P. A., van Loon, R. E., Veldman, M., Kusmallah, N., Ekster, P. J. C, & Mooren, T. (2021). Overcoming barriers to mental health care: Multimodal traumafocused treatment approach for unaccompanied refugee minors*. Child and Adolescent Psychiatry and Mental Health*, *15*(1). <https://doi.org/10.1186/s13034-021-00404-3>

Vickers, B. (2005). Cognitive model of the maintenance and treatment of post-traumatic stress disorder applied to children and adolescents. *Clinical Child Psychology and Psychiatry*, *10*(2), 217–234. <https://doi.org/10.1177/1359104505051212>

Vossoughi, N., Jackson, Y., Gusler, S., & Stone, K. (2018). Mental Health Outcomes for Youth Living in Refugee Camps: A Review. *Trauma, Violence, & Abuse*, *19*(5), 528–542. <https://doi.org/10.1177/1524838016673602>

Wichmann, M. L.-Y., Pawils, S., Richters, J., & Metzner, F. (2022). School-Based Interventions for Child and Adolescent Victims of Interpersonal Violence. *Trauma, Violence, & Abuse*. <https://doi.org/10.1177/15248380221078892>

Yayan, Düken, M. E., Özdemir, A. A., & Çelebioğlu, A. (2020). Mental Health Problems of Syrian Refugee Children: Post-Traumatic Stress, Depression and Anxiety. *Journal of Pediatric Nursing*, *51*, e27–e32. <https://doi.org/10.1016/j.pedn.2019.06.012>
