# Supplementary material for: Acculturation and Culturally Adapted CBT for Refugee Youth and Families—A Systematic Review
Source: J Child Adolesc Trauma. 2026 Jul 28;19(4):2193–214. doi: 10.1007/s40653-026-00947-7 (PMC13737252; doi:10.1007/s40653-026-00947-7)
Supplement: Supplementary file 2 [file 40653_2026_947_MOESM2_ESM.doc]

**For the attention of Dominic McSherry, PhD, School of Psychology, Ulster University, Coleraine, Northern Ireland, UK**

Editor-in-Chief of *Journal of Child & Adolescent Trauma*

7 April 2026

Dear Dominic McSherry,

Re: **Acculturation and Culturally Adapted CBT for Refugee Youth and Families—A Systematic Review**

Thank you for the constructive comments from the reviewers and opportunity to revise and resubmit our manuscript “Acculturation and Culturally Adapted CBT for Refugee Youth and Families—A Systematic Review.” We have revised the manuscript based on the reviewers’ comments and addressed each of the comments as detailed on the following pages.

Neither the manuscript nor any significant part of it is under consideration for publication elsewhere or has appeared elsewhere in a manner that could be construed as a prior or duplication of the same work.

We look forward to hearing from you.

Many thanks.

Kind Regards,

Authors

**Reviewers’ Comments and Responses From Authors**

Reviewer #1:

1. Reviewer Comment: Thank you for the opportunity to review your manuscript. This manuscript addresses a highly important topic, particularly the role of acculturation and culturally adapted CBT for refugee youth and families. You have incorporated the necessary clarifications, and overall, the manuscript is now in strong shape to proceed. At this point, my main recommendation is to carefully proofread the paper for spelling errors and conduct a final round of substantive editing to improve clarity, coherence, and flow. These refinements will enhance the readability and overall quality of the manuscript. However, these can be done later during the publication process.
   a. Response: Thank you very much for your positive feedback regarding the introduction. We are pleased that you find the manuscript is now in strong shape to proceed. We have proofread and conducted further editing to enhance clarity, coherence, and overall flow, as recommended and will continue to do so throughout the rest of the publication process. We appreciate your guidance and support throughout the review process.

Reviewer #2:

1. Reviewer Comment: This article is clearly written and presents a strong and thoughtfully executed methodology. One area that may benefit from clarification is the secondary literature search strategy. It appears that no studies were identified through supplemental methods (e.g., citation chaining or snowball searches). While this can be inferred by the reader, explicitly stating this would enhance transparency and methodological rigor. I also commend the authors for focusing on effective, evidence-based interventions for refugee populations, an area of critical clinical and public health importance.
   a. Response: Thank you very much for your feedback and for raising this point regarding clarifying our secondary literature search strategy. We have updated this section and now explicitly state that none of the studies included in this review were identified through supplemental methods (e.g., citation chaining, snowball searches).
2. Reviewer Comment: That said, I have concerns that a substantial body of relevant literature may have been overlooked. Based on the authors' definition of culturally adapted cognitive behavioral therapy (CA-CBT), it seems likely that trauma-focused cognitive behavioral therapy (TF-CBT) studies should have been considered. Cultural sensitivity is a foundational and integral component of TF-CBT, rather than an adjunctive feature. TF-CBT explicitly emphasizes attention to cultural values, linguistic needs, family context, and meaning-making, all of which are central to effective treatment with refugee and culturally diverse youth (Cohen et al., 2017; Deblinger et al., 2016). As both a TF-CBT provider and researcher, I note that the model inherently prioritizes cultural responsiveness through flexible implementation, caregiver involvement, and collaborative goal-setting. The inclusion of caregivers or other safe adults is a core element of TF-CBT and serves not only to reinforce skill generalization outside of sessions, but also to ensure that treatment aligns with the family's cultural values, traditions, and belief systems (Cohen et al., 2017). These components appear highly consistent with the aims of CA-CBT as described in the current review. It would therefore strengthen the manuscript to more explicitly address why TF-CBT studies were excluded from this review.
   a. Response: Thank you for identifying this issue. We have revised our manuscript to address this concern and added to the limitations section to clearly address why some TF-CBT studies were excluded from this review. We have also included that it would be beneficial for future reviews and analyses to broaden the scope to acculturation-informed TF-CBT studies to ensure relevant literature is not overlooked. The limitation section now includes: “Another limitation is that, given how cultural sensitivity is a core aspect of TF-CBT, TF-CBT might be considered as inherently qualifying as CA-CBT under the authors’ definition. TF-CBT is considered culturally responsive due to its flexibility, collaborative nature, and inclusion of family and caregivers (Cohen et al., 2018; Deblinger et al., 2016). This can help with ensuring the treatment process is in accord with the client’s principles, beliefs, cultural norms, language, etc. (Cohen et al., 2018; Deblinger et al., 2016). Therefore, it could be argued that TF-CBT falls under the authors' description of CA-CBT. However, this review aimed to examine CBT interventions that included specific cultural adaptations to treatment, taking into account factors such as the client’s cultural background, values, and language (Bernal et al., 2009; Hinton et al., 2012; Taylor et al., 2022). Therefore, while TF-CBT is inherently culturally responsive and flexible, only studies that included TF-CBT interventions with specific cultural adaptations were considered to qualify as CA-CBT interventions in this review. That being said, given the substantial overlap between CA-CBT and TF-CBT, it would be beneficial for future reviews and analyses to broaden the scope to acculturation-informed TF-CBT studies. Additionally, the included studies did not compare acculturation-informed CA-CBT with CA-CBT, traditional CBT, or TF-CBT not informed by acculturation. Therefore, it would also be worthwhile for future reviews and analyses to compare the acculturation-informed CA-CBT studies included in this review with the CA-CBT interventions displayed in Table 4.”
3. Reviewer Comment: Additionally, the discussion could be enhanced by examining specific principles that may further optimize the effectiveness of CA-CBT, such as caregiver involvement, group versus individual treatment formats, and treatment delivery context (e.g., in-person versus virtual settings). Providing concrete examples of how interventions were culturally adapted or acculturation-informed—such as modifications to language, metaphors, session structure, or engagement strategies—would also improve clinical applicability and conceptual clarity. Overall, addressing these points would enhance the comprehensiveness of the review and further strengthen its contribution to the literature on culturally responsive trauma interventions for refugee youth.
   a. Response: Thank you very much for raising this. We have revised the discussion section to more clearly examine specific principles that are relevant to clinical practice and provide concrete examples of how interventions were culturally adapted and acculturation-informed. Specifically, the “Research and Clinical Implications” subsection of the discussion section has been substantially updated to address this concern.
